# Expression of S-adenosylmethionine Hydrolase in Tissues Synthesizing Secondary Cell Walls Alters Specific Methylated Cell Wall Fractions and Improves Biomass Digestibility

**DOI:** 10.3389/fbioe.2016.00058

**Published:** 2016-07-19

**Authors:** Aymerick Eudes, Nanxia Zhao, Noppadon Sathitsuksanoh, Edward E. K. Baidoo, Jeemeng Lao, George Wang, Sasha Yogiswara, Taek Soon Lee, Seema Singh, Jenny C. Mortimer, Jay D. Keasling, Blake A. Simmons, Dominique Loqué

**Affiliations:** ^1^Joint BioEnergy Institute, Emeryville, CA, USA; ^2^Biological Systems and Engineering Division, Lawrence Berkeley National Laboratory, Berkeley, CA, USA; ^3^Department of Bioengineering, University of California, Berkeley, CA, USA; ^4^Department of Chemical & Biomolecular Engineering, University of California, Berkeley, CA, USA; ^5^Department of Chemical Engineering, Conn Center for Renewable Energy, University of Louisville, Louisville, KY, USA; ^6^Sandia National Laboratory, Livermore, CA, USA; ^7^Université Claude Bernard Lyon 1, INSA de Lyon, CNRS, UMR5240, Microbiologie, Adaptation et Pathogénie, Villeurbanne, France

**Keywords:** cell wall, lignin, S-adenosylmethionine, AdoMetase, Yang cycle, glucuronoxylan, saccharification

## Abstract

Plant biomass is a large source of fermentable sugars for the synthesis of bioproducts using engineered microbes. These sugars are stored as cell wall polymers, mainly cellulose and hemicellulose, and are embedded with lignin, which makes their enzymatic hydrolysis challenging. One of the strategies to reduce cell wall recalcitrance is the modification of lignin content and composition. Lignin is a phenolic polymer of methylated aromatic alcohols and its synthesis in tissues developing secondary cell walls is a significant sink for the consumption of the methyl donor S-adenosylmethionine (AdoMet). In this study, we demonstrate in *Arabidopsis* stems that targeted expression of AdoMet hydrolase (AdoMetase, E.C. 3.3.1.2) in secondary cell wall synthesizing tissues reduces the AdoMet pool and impacts lignin content and composition. In particular, both NMR analysis and pyrolysis gas chromatography mass spectrometry of lignin in engineered biomass showed relative enrichment of non-methylated *p*-hydroxycinnamyl (H) units and a reduction of dimethylated syringyl (S) units. This indicates a lower degree of methylation compared to that in wild-type lignin. Quantification of cell wall-bound hydroxycinnamates revealed a reduction of ferulate in AdoMetase transgenic lines. Biomass from transgenic lines, in contrast to that in control plants, exhibits an enrichment of glucose content and a reduction in the degree of hemicellulose glucuronoxylan methylation. We also show that these modifications resulted in a reduction of cell wall recalcitrance, because sugar yield generated by enzymatic biomass saccharification was greater than that of wild-type plants. Considering that transgenic plants show no important diminution of biomass yields, and that heterologous expression of AdoMetase protein can be spatiotemporally optimized, this novel approach provides a valuable option for the improvement of lignocellulosic biomass feedstock.

## Introduction

**Figure 1:**
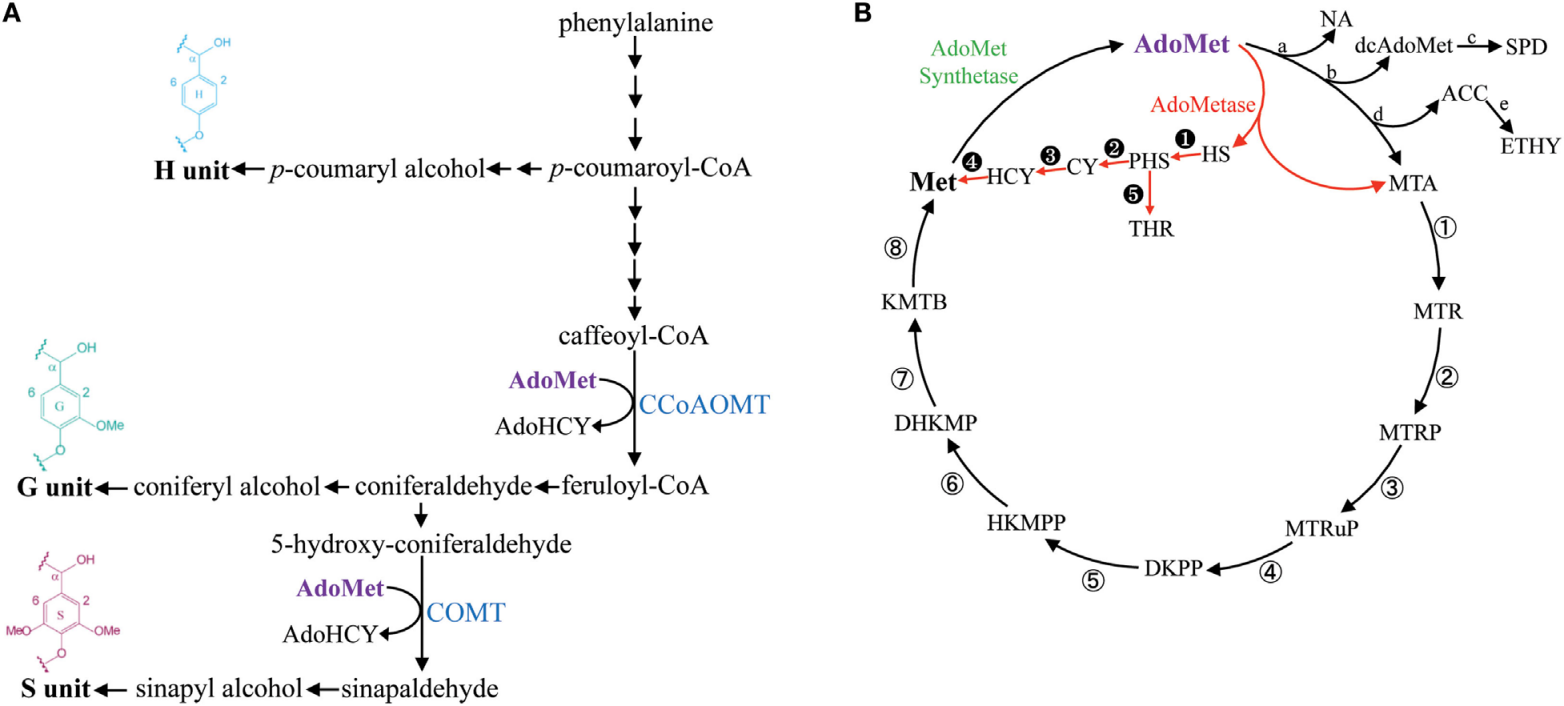
**Simplified lignin biosynthetic pathway and the methionine salvage cycle (or Yang cycle)**. Enzymatic steps consuming AdoMet in the lignin biosynthetic pathway are catalyzed by CCoAOMT and COMT **(A)**. The metabolic shunt mediated by AdoMetase in the Yang cycle is shown in red **(B)**. Abbreviations: ACC, 1-aminocyclopropanecarboxylate; AdoHCY, S-adenosylhomocysteine; AdoMet, S-adenosylmethionine; CCoAOMT, caffeoyl CoA *O*-methyltransferase; COMT, caffeic acid *O*-methyltransferase; CY, cystathionine; dcAdoMet, decarboxylated AdoMet; DHKMP, 1,2-dihydroxy-3-keto-5-methylthiopentene; DKPP, 2,3-diketo-5-methylthiopentyl-1-phosphate; ETHY, ethylene; HCY, homocysteine; HKMPP, 2-hydroxy-3-keto-5-methylthiopentenyl-1-phosphate; HS, homoserine; KMTB, α-ketomethylthiobutyrate; Met, methionine; MTA, methylthioadenosine; MTR, 5-methylthioribose; MTRP, 5-methylthioribose-1-phosphate; MTRuP, methylthioribulose-1-phosphate; NA, nicotianamide; PHS, *O*-phosphohomoserine; SPD, spermidine; THR, threonine. a, NA synthase; b, AdoMet decarboxylase; c, SPD synthase; d, ACC synthase; e, ACC oxidase; ①, MTA nucleosidase; ②, MTR kinase; ③, MTRP isomerase; ④, MTRuP dehydratase; ⑤, DKPP enolase; ⑥, HKMPP phosphatase; ⑦, DHKMP dioxygenase; ⑧, KMTB aminotransferase. ➊ HS kinase; ➋, CY synthase; ➌, CY β-lyase; ➍, Met synthase; ➎, Thr synthase.

Lignin is a phenolic polymer produced by oxidative polymerization of methylated hydroxycinnamyl alcohols (or monolignols) synthesized from phenylalanine (Figure 1A). Guaiacyl (G) units are derived from coniferyl alcohol, which contains one methyl group, and syringyl (S) units are derived from dimethylated sinapyl alcohol (Figure 1A). G and S units are the most common lignin monomers in angiosperms, whereas *p*-hydroxyphenyl (H) units derived from the polymerization of non-methylated *p*-coumaryl alcohol are typically less abundant (Boerjan et al., 2003). The enzymatic saccharification of lignocellulosic biomass for the production of fermentable sugars is negatively impacted by the presence of lignin and, consequently, several strategies have been suggested to overcome lignin recalcitrance and to control its spatiotemporal deposition in bioenergy crops (Chen and Dixon, 2007; Eudes et al., 2014; Mottiar et al., 2016).

S-adenosylmethionine (AdoMet) is a key intermediate in one-carbon metabolism that serves as a universal methyl-group donor for the methylation of a large number of metabolites (Hanson and Roje, 2001). In addition to being used as a substrate by AdoMet-utilizing methyltransferases, it also acts as a precursor in the synthesis of polyamines, nicotianamine, phytosiderophores, 5′-deoxyadenosyl radicals, and ethylene (Roje, 2006). As part of the methionine salvage cycle (or Yang cycle), AdoMet is synthesized from methionine (Met) by AdoMet synthetase (Figure 1B) (Albers, 2009).

The synthesis of lignin in tissues producing secondary cell walls (SCWs) is associated with a massive demand for one-carbon units and requires large supply and efficient recycling of AdoMet (Hanson and Roje, 2001; Amthor, 2003). The lignin biosynthetic pathway contains two enzymes that consume AdoMet for transmethylation reactions: Caffeoyl CoA *O*-methyltransferase (CCoAOMT) is involved in the first methylation step required for the production of G units, and caffeic acid *O*-methyltransferase (COMT) performs the second methylation step needed for the synthesis of S units (Figure 1A) (Zhong et al., 1998; Osakabe et al., 1999; Li et al., 2000; Guo et al., 2001). Moreover, SCWs contain the hemicellulose 4-*O*-methylglucuronoxylan (GX), which also requires an important supply of AdoMet for synthesis (Scheller and Ulvskov, 2010). In particular, in *Arabidopsis*, three DUF579 domain-containing methyltransferases act redundantly for the 4-*O*-methylation of glucuronic acid (GlcA) side chains on GX (Lee et al., 2012; Urbanowicz et al., 2012; Yuan et al., 2014).

The importance of AdoMet as a methyl donor for lignin biosynthesis has been illustrated by several mutant studies in *Arabidopsis* and maize. For example, mutation in one of the AdoMet synthetases (AdoMetS3) in *Arabidopsis* results in concomitant reductions of AdoMet synthetase activity, AdoMet pools, and lignin content (Shen et al., 2002). Recently, it was shown that mutations in genes responsible for the synthesis of 5-methyltetrahydrofolate, which is used as methyl donor by Met synthase for the production of Met from homocysteine, leads to reductions of lignin content in maize and *Arabidopsis* (Tang et al., 2014; Li et al., 2015; Srivastava et al., 2015). These mutations affect methylenetetrahydrofolate reductase (MTHFR) or folylpolyglutamate synthase (FPGS), and reductions in both pools of AdoMet and its precursor Met were measured in the *Arabidopsis fpgs* mutant (Srivastava et al., 2011, 2015). Importantly, the *Arabidopsis fpgs* mutant is affected in a FPTGS isoform preferentially expressed in vascular tissues and does not show any defects in above-ground biomass yield (Srivastava et al., 2015).

In this study, we evaluated in *Arabidopsis* the impact of expressing S-adenosylmethionine hydrolase (AdoMetase) in tissues producing SCWs. The *AdoMetase* gene has been cloned from the coliphage T3 (Hughes et al., 1987) and its product hydrolyzes AdoMet into homoserine and methylthioadenosine, which creates a metabolic shunt within the Yang cycle (Figure 1B). Previous genetic engineering studies have demonstrated the efficacy of expressing AdoMetase stage-specifically in climacteric fruits to reduce ethylene production from AdoMet and slow the ripening process (Good et al., 1994; Mathews et al., 1995; Clendennen et al., 1999). We used, in this study, the promoter of a SCW cellulose synthase (*pAtIRX5*) to drive the expression of AdoMetase in stem interfascicular fibers and xylem vessels in which the biosynthesis of both lignin and GX requires significant amounts of AdoMet. Focusing our analyses on the main SCW components, we demonstrate that targeting the expression of the AdoMetase protein in SCW-producing tissues reduces the content of lignin and its degree of methylation, presumably by affecting simultaneously both methylation steps of the lignin biosynthetic pathway. We also show that biomass from engineered plants is characterized by an enrichment in glucose content, reduction of *O*-methylated GlcA residues on GX polymer, and lower amount of cell wall-bound ferulate. Although the transgenic plants show a reduction in stem size, their biomass yields are similar to those of wild-type plants, while their release of sugars from biomass upon enzymatic treatment is enhanced.

## Materials and Methods

### Plant Material and Growth Conditions

*Arabidopsis thaliana* (ecotype Columbia, Col-0) seeds were germinated directly on soil. Growing conditions were 150 μmol/m^2^/s, 22°C, 60% humidity, and 10 h of light per day. Selection of T2 and identification of T3 homozygous transgenic plants was made on Murashige and Skoog vitamin medium (PhytoTechnology Laboratories, Shawnee Mission, KS, USA), supplemented with 1% sucrose, 1.5% agar, and 25 μg/mL hygromycin.

### *pAtIRX5:AdoMetase* Construct and Plant Transformation

To generate the binary vector pA6-*pAtIRX5:AdoMetase*, the *pAtIRX5* promoter described in Eudes et al. (2012) was released from pCR™-Blunt vector (Life Technologies, Foster City, CA, USA) using *Kpn*I/*Nhe*I restriction enzymes and ligated into the pA6-GW binary vector harboring a gateway cloning cassette (Yang et al., 2013) and digested with *Kpn*I and *Avr*II (*Nhe*I compatible site) restriction enzymes to produce the pA6-*pAtIRX5-*GW binary vector. A nucleotide sequence encoding AdoMetase from the enterobacteria phage T3 (UniProtKB/Swiss-Prot accession number P07693.1) flanked with the Gateway *att*B1 (5′-end) and attB2 (3′-end) recombination sites was synthesized for expression in *Arabidopsis* (Data S1 in Supplementary Material) (GenScript, Piscatway, NJ, USA) and cloned into the Gateway pDONR221-P1P2 entry vector by BP recombination (Life Technologies, Foster City, CA, USA). An entry clone was LR recombined with the pA6-*pAtIRX5*-GW vector to generate the pA6-*pAtIRX5*:*AdoMetase* construct. The construct was introduced into wild-type *Arabidopsis* plants (ecotype Col-0) via *Agrobacterium tumefaciens*-mediated transformation (Bechtold and Pelletier, 1998).

### RNA Extraction and RT-PCR

Total RNA (1 μg) was extracted from stems of 5-week-old wild-type and T3 homozygous transgenic lines using the Plant RNeasy extraction kit (Qiagen, Valencia, CA, USA) and reverse-transcribed using the Transcriptor First Strand cDNA Synthesis Kit (Roche Applied Science, Indianapolis, IN, USA). The cDNA preparations obtained were quality-controlled using the *tub8*-specific oligonucleotides Tub8-fw and Tub8-rv (Table S1 in Supplementary Material), and the *AdoMetase* transcripts were detected using the oligonucleotides AdoMetase-fw and AdoMetase-rv (Table S1 in Supplementary Material).

### Metabolites Extraction

*Arabidopsis* stems of 5-week-old wild-type and T3 homozygous *pAtIRX5:AdoMetase* lines were collected in liquid nitrogen and stored at −80°C until further utilization. Collected stems were pulverized in liquid nitrogen and metabolites were extracted as previously described (Van de Poel et al., 2010): 100–200 mg of frozen stem powder was homogenized with 1 mL of trichloroacetic acid (5% w/v) and mixed (1,400 rpm) for 15 min at 4°C. Extracts were cleared by centrifugation (10 min, 20,000 × *g*, at 4°C) and filtered using Amicon Ultra centrifugal filters (3,000 Da MW cutoff regenerated cellulose membrane; EMD Millipore, Billerica, MA, USA). Filtered extracts were kept at −20°C until LC–MS analysis.

### Cell Wall-Bound Hydroxycinnamates Extraction

The biomass from senesced wild-type plants and T3 homozygous *pAtIRX5:AdoMetase* lines was used to measure cell wall-bound ferulate and *p*-coumarate as previously described (Eudes et al., 2015). Extracted biomass (10 mg) was mixed with 500 μL of 2M NaOH and shaken at 1,400 rpm for 24 h at 30°C. The mixture was acidified with 100 μL of concentrated HCl (12N), and subjected to three ethyl acetate partitioning steps. Ethyl acetate fractions were pooled, dried *in vacuo*, and suspended in 50% (v/v) methanol–water prior to LC–MS analysis.

### Biomass Compositional Analysis

The biomass from senesced wild-type plants and T3 homozygous *pAtIRX5:AdoMetase* lines was used for analysis. Biomass was extracted sequentially by sonication (20 min) with 80% (v/v) ethanol–water (three times), 100% acetone (one time), chloroform–methanol (1:1, v/v, one time), and 100% acetone (one time). The standard NREL protocol consisting of a two-step acid hydrolysis of biomass was used to measure lignin content and determine monosaccharide composition (Sluiter et al., 2008). Hydrolysis of biomass with trifluoroacetic acid was performed as previously described (Eudes et al., 2012) for the release of glucose residues that are not polymerized into crystalline cellulose. The chemical composition of lignin was analyzed by pyrolysis-gas chromatography (GC)/mass spectrometry (MS) using a previously described method (Eudes et al., 2012). Lignin pyrolysis products were identified by comparing their mass spectra with those of the NIST library and those previously reported (Ralph and Hatfield, 1991; Del Río and Gutiérrez, 2006).

### LC–MS Analysis

High-performance liquid chromatography (HPLC) mobile phases were composed of HPLC grade solvents. AdoMet, homoserine, methylthioadenosine, homocysteine, Met, and threonine were analyzed using HPLC, electrospray ionization (ESI), and time-of-flight (TOF) MS as previously described in Bokinsky et al. (2013). Ferulate and *p*-coumarate were analyzed using HPLC–ESI–TOF–MS as previously described (Eudes et al., 2013). 4-*O*-MeGlcA from biomass hydrolyzates was analyzed and quantified by HPLC–ESI–TOF–MS. The separation of MeGlcA was conducted on a Carbomix H-NP5 column with 8% cross linkage (150 mm length, 4.6 mm internal diameter, and 5 μm particle size; Sepax Technologies, DE, USA) using an Agilent Technologies 1200 Series Rapid Resolution HPLC system. The temperature of the sample tray was maintained at 6°C by an Agilent FC/ALS Thermostat. The column compartment was set to 50°C. A sample injection volume of 5 μL was used. Metabolites were eluted isocratically with a mobile phase composed of 0.1% formic acid in water. A flow rate of 0.25 mL/min was used throughout. The HPLC system was coupled to an Agilent Technologies 6210 TOF mass spectrometer by a 1/4 post-column split. Contact between both instrument set-ups was established by a LAN card to trigger the MS into operation upon the initiation of a run cycle from the MassHunter workstation (Agilent Technologies, CA, USA). Nitrogen was used as both the nebulizing and drying gas to facilitate the production of gas-phase ions. Drying and nebulizing gases were set to 11 L/min and 30 psi, respectively, and a drying gas temperature of 330°C was used throughout. ESI was conducted in the negative ion mode and a capillary voltage of −3,500 V was utilized. MS experiments were carried out in the full scan mode, at 0.86 spectra/second, for the detection of [M-H]^−^ ions. The instrument was tuned for a range of 50–1700 m/z and ions were acquired from 100 to 1000 m/z. Prior to LC–TOF–MS analysis, the TOF–MS was calibrated via an ESI-L low concentration tuning mix (Agilent Technologies, CA, USA). Data acquisition and processing were performed by the MassHunter software package. A 4-*O*-MeGlcA authentic standard was obtained from LC Scientific Inc. (Concord, Canada). All metabolites were quantified via calibration curves of authentic standard compounds for which the *R*^2^ coefficients were ≥0.99.

### HPAEC-PAD Analysis

Except for the quantification of 4-*O*-MeGlcA for which LC–MS analysis was used because of higher sensitivity (see Materials and Methods above), the monosaccharide composition of hydrolyzed biomass was determined by HPAEC-PAD. Measurements of fucose, rhamnose, arabinose, galactose, glucose, galacturonic acid, and GlcA contents were conducted as previously described (Eudes et al., 2012). Because this method cannot separate xylose from mannose, a different HPAEC-PAD method was used for the separation and measurement of these two monosaccharides: the chromatography was performed on a PA20 column (Dionex, Sunnyvale, CA, USA) at a flow rate of 0.4 mL min^−1^ and the column oven set at 30°C. Before injection of each sample (20 μL), the column was washed with 200 mM NaOH for 10 min and equilibrated with 4 mM NaOH for 5 min. The elution program was as follows: 6 min with 4 mM NaOH, ramp down to 1 mM NaOH for 2 min; 11 min at 1 mM NaOH, ramp to 450 mM NaOH for 6 s; then 450 mM NaOH for 18 min. Monosaccharides were detected using a pulsed amperometric detector (gold electrode) set on waveform A according to manufacturer’s instructions. A calibration curve of monosaccharide standards that includes l-fucose, l-rhamnose, l-arabinose, d-galactose, d-glucose, d-xylose, d-mannose, d-galacturonic acid, and d-GlcA (Sigma-Aldrich, St Louis, MO, USA) was run for verification of response factors.

### 2D ^13^C-^1^H Heteronuclear Single Quantum Coherence NMR Spectroscopy

Stem material from wild-type and *pAtIRX5:AdoMetase* lines was extracted and ball milled as previously described (Kim and Ralph, 2010; Mansfield et al., 2012). The gels were formed using DMSO-d_6_/pyridine-d_5_ (4:1) and sonicated until homogeneous in a Branson 2510 table-top cleaner (Branson Ultrasonic Corporation, Danbury, CT, USA). The homogeneous solutions were transferred to NMR tubes. Heteronuclear Single Quantum Coherence (HSQC) spectra were acquired at 25°C using a Bruker Avance-600 MHz instrument equipped with a 5 mm inverse-gradient ^1^H/^13^C cryoprobe using a hsqcetgpsisp2.2 pulse program (ns = 400, ds = 16, number of increments = 256, d_1_ = 1.0 s) (Heikkinen et al., 2003). Chemical shifts were referenced to the central DMSO peak (δ_C_/δ_H_ 39.5/2.5 ppm). Assignment of the HSQC spectra was described elsewhere (Yelle et al., 2008; Kim and Ralph, 2010). A semi-quantitative analysis of the volume integrals of the HSQC correlation peaks was performed using Bruker’s Topspin 3.1 (Windows) processing software. A Gaussian apodization in F_2_ (LB = −0.50, GB = 0.001) and squared cosine-bell in F_1_ (LB = −0.10, GB = 0.001) were applied prior to 2D Fourier transformation.

### Cell Wall Pretreatments and Saccharification

Ball-milled senesced stems (10 mg) were mixed with 340 μL of NaOH (0.25%, w/v), shaken at 1,400 rpm (30°C, 30 min), and autoclaved at 120°C for 1 h. Saccharification was initiated by adding 650 μL of 100 mM sodium citrate buffer pH 5 containing 80 μg/mL tetracycline and 1% w/w Cellic CTec2 cellulase (Novozymes, Davis, CA, USA). After 48 h of incubation at 50°C with shaking (800 rpm), samples were centrifuged (20,000 × *g*, 3 min) and 10 μL of the supernatant was collected for measurement of reducing sugars using the 3,5-dinitrosalicylic acid assay and glucose solutions as standards (Miller, 1959).

## Results

### Expression of AdoMetase in *Arabidopsis*

**Figure 2:**
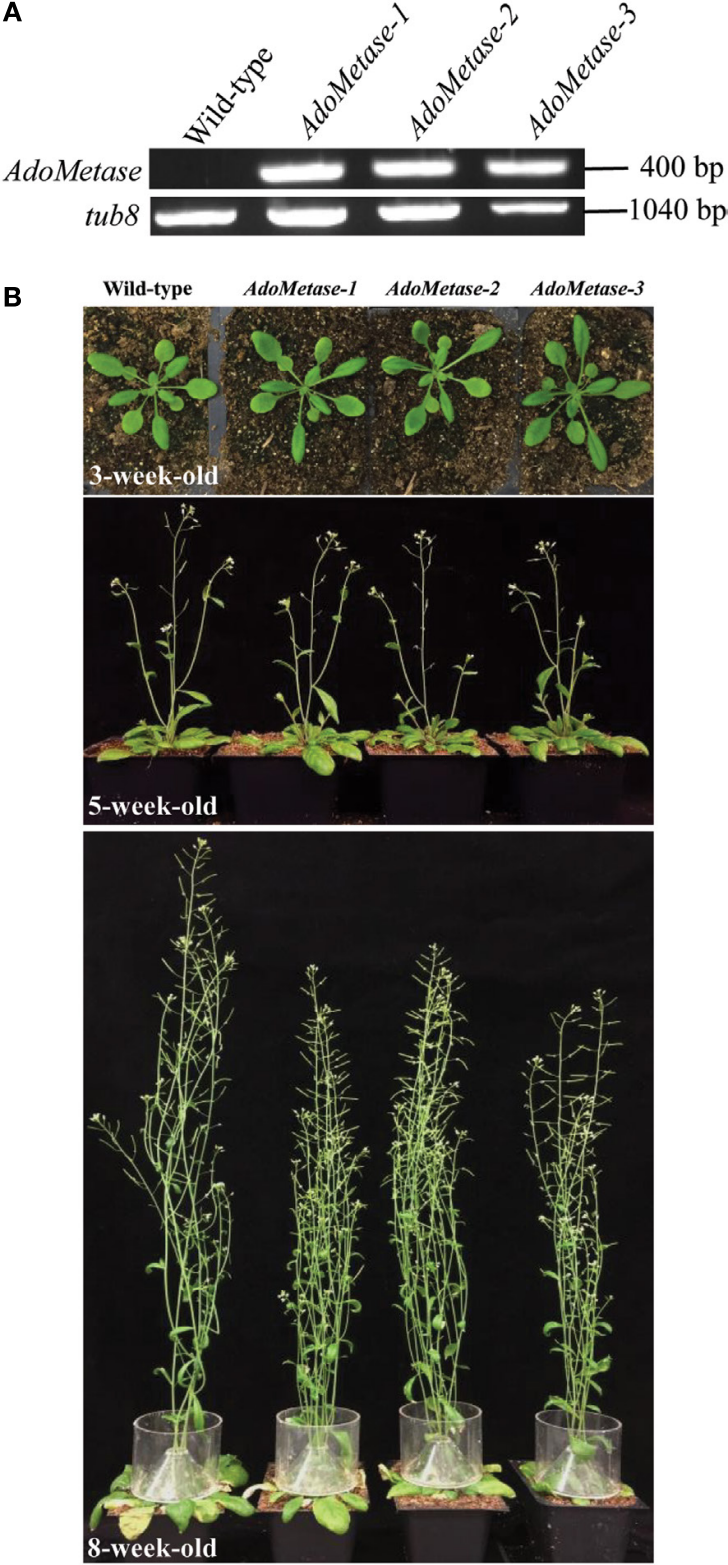
***AdoMetase* expression in *pAtIRX5:AdoMetase* lines and phenotype**. **(A)** *AdoMetase* transcripts were detected by RT-PCR using stem mRNA from three independent 5-week-old T3 homozygous *pAtIRX5:AdoMetase* (*AdoMetase*) transformants. cDNA synthesized from stem mRNA of wild-type plants were used as a negative control. *Tub8*-specific primers were used to assess cDNA quality for each sample. **(B)** Comparison of the growth and development of wild-type and *pAtIRX5:AdoMetase* (*AdoMetase*) lines at different stages. Upper panel: 3-week-old rosette; middle panel: 5-week-old flowering stage; bottom panel: 8-week-old senescing stage.

The promoter of the SCW cellulose synthase gene *AtCesA4* (*pAtIRX5*), which is specifically active in interfascicular fibers and xylem vessels (Eudes et al., 2012), was selected to express specifically the *AdoMetase* protein in *Arabidopsis* stems. Reverse transcription PCR (RT-PCR) using mRNA from stems of three independent homozygous transformants confirmed *AdoMetase* expression (Figure 2A). These lines are fertile, and, compared to wild type, show neither obvious growth defect phenotype nor a decrease in biomass yield of total stems, but exhibit a 12–20% height reduction of the main stem (Figure 2B; Table 1).

**Table 1:** **Height of the main inflorescence stem and total stem dry weight of senesced mature wild-type and *pAtIRX5:AdoMetase* (*AdoMetase*) lines**.

Plant line	Height (cm)	Dry weight (mg)
Wild type	62.3 ± 2.4	263.9 ± 21.4
*AdoMetase-1*	53.8 ± 2.1*	246.2 ± 26.3
*AdoMetase-2*	55.1 ± 1.0*	277.6 ± 20.3
*AdoMetase-3*	50.2 ± 71.7*	234.6 ± 38.7

Values are means ± SE from six biological replicates (*n* = 6). Asterisks indicate significant differences from the wild type using the unpaired Student’s *t*-test (*P < 0.05).

### Metabolite Analysis of *pAtIRX5: AdoMetase* Lines

Metabolites from stems of the *pAtIRX5:AdoMetase* lines were extracted and analyzed using LC–MS (Table 2). Compared to wild type, AdoMet content was reduced by 38–51% in transgenic plants, and the AdoMetase activity products homoserine and methyltioadenosine were detected only in stems of *pAtIRX5:AdoMetase* lines. These results confirm the activity of AdoMetase in *Arabidopsis* stems and validate its use to reduce the AdoMet pool. Similarly, homocysteine was detected only in transgenic plants. The latter could be the result of higher homoserine conversion via *O*-phosphohomoserine and cystathionine (Figure 1B), which is supported with higher amounts of threonine (threefold) measured in stems of *pAtIRX5:AdoMetase* plants. Measurement of Met showed unchanged content in two lines and a 1.9-fold increase in the third one.

**Table 2:** **Quantitative analysis of metabolites in stems from 5-week-old wild-type and *pAtIRX5:AdoMetase* (*AdoMetase*) lines**.

Mean ± SE (nmole g^−1^ fresh weight)	Plant line			
	Wild type	*AdoMetase-1*	*AdoMetase-2*	*AdoMetase-3*
AdoMet	18.5 ± 0.7	11.5 ± 1.1*	9.5 ± 1.1*	9.1 ± 0.7*
Homoserine	nd	13.0 ± 2.7	12.5 ± 1.4	10.7 ± 1.3
Methylthioadenosine	nd	2.2 ± 0.2	2.2 ± 0.1	2.3 ± 0.1
Homocysteine	nd	25.2 ± 2.5	11.9 ± 3.0	20.9 ± 5.2
Threonine	551 ± 67	1628 ± 93*	1681 ± 131*	1634 ± 109*
Methionine	25.7 ± 3.3	23.7 ± 1.3	24.7 ± 2.6	49.2 ± 5.6*

Values are means ± SE from six biological replicates (*n* = 6). nd, Not detected. Asterisks indicate significant differences from the wild type using the unpaired Student’s *t*-test (*P < 0.005).

Cell wall-bound *p*-coumarate and ferulate released from cell walls by mild alkaline hydrolysis were also analyzed using LC–MS (Table 3). The content of *p*-coumarate was unchanged in the *pAtIRX5:AdoMetase* lines, whereas ferulate was reduced by 22–24%.

**Table 3:** **Quantitative analysis of cell wall-bound ferulate and *p*-coumarate in stems from senesced mature dried wild-type and *pAtIRX5:AdoMetase* (*AdoMetase*) lines**.

Plant line	Mean ± SE (μg g^−1^ dry weight)	
	Ferulate	*p*-Coumarate
Wild type	15.2 ± 0.7	23.9 ± 3.8
*AdoMetase-1*	11.7 ± 0.8*	23.7 ± 5.0
*AdoMetase-2*	11.8 ± 0.8*	24.6 ± 3.5
*AdoMetase-3*	11.5 ± 0.6*	23.3 ± 1.4

Values are means ± SE from four biological replicates (*n* = 4). Asterisks indicate significant differences from the wild type using the unpaired Student’s *t*-test (*P < 0.05).

### Lignin Content and Monomeric Composition in *pAtIRX5:AdoMetase* Lines

**Figure 3:**
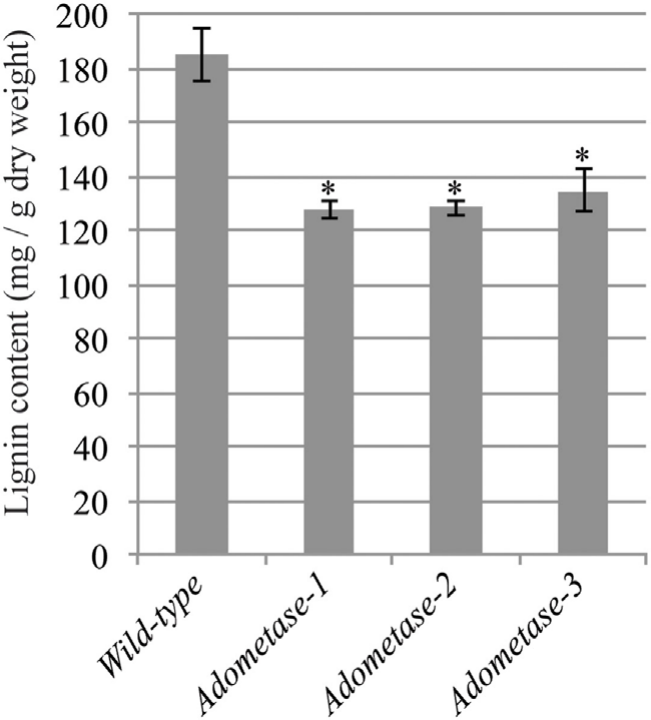
**Lignin content in senesced mature stems from wild-type and *pAtIRX5:AdoMetase* (*AdoMetase*) lines**. Values are means ± SE from four biological replicates (*n* = 4). Asterisks indicate significant differences from the wild type using the unpaired Student’s *t*-test (*P < 0.01).

The Klason method was used to measure lignin content and revealed reductions ranging from 27 to 31% in stems of the *pAtIRX5:AdoMetase* lines compared to wild type (Figure 3). Cell wall material from stems of wild-type and *pAtIRX5:AdoMetase* lines was analyzed by pyrolysis-GC*/*MS for the determination of the lignin monomer composition. For each line, identification and relative quantification of the pyrolysis products derived from H, G, or S units allowed determination of H/G/S ratios (Table 4, Table S1 in Supplementary Material). In transgenic plants, the relative amount of G units is unchanged, whereas that of H and S units is increased by 2.5–2.8-fold and reduced by ~1.4-fold, respectively.

**Table 4:** **Lignin monomeric composition in senesced mature stems from wild-type and *pAtIRX5:AdoMetase* (*AdoMetase*) lines**.

	%H	%G	%S	S/G
Wild type	3.2 ± 0.4	67.1 ± 1.2	29.7 ± 1.1	0.44
*AdoMetase-1*	8.2 ± 0.3*	70.7 ± 1.2	21.1 ± 1.1*	0.30*
*AdoMetase-2*	9.1 ± 1.1*	70.9 ± 1.1	20.0 ± 1.4*	0.28*
*AdoMetase-3*	8.1 ± 0.8*	71.1 ± 1.3	20.7 ± 1.5*	0.29*

Values are means ± SE from four biological replicates (*n* = 4). Asterisks indicate significant differences from the wild-type using the unpaired Student’s *t*-test (*P < 0.005).

**Figure 4:**
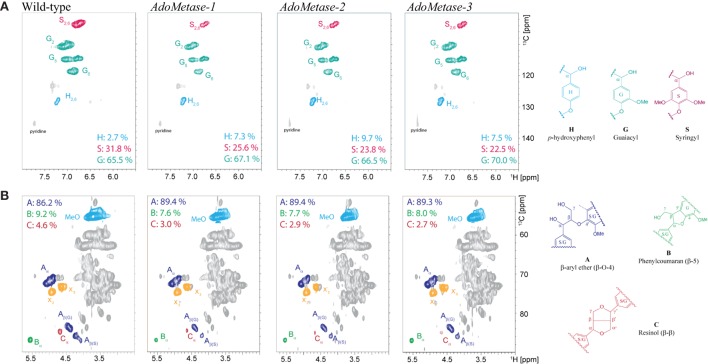
**Lignin composition and interunit linkages in senesced mature stems from wild-type and *pAtIRX5:AdoMetase* (*AdoMetase*) lines**. The aromatic **(A)** and aliphatic **(B)** regions of partial short-range 13C–1H (HSQC) spectra of cell wall material are shown. Lignin monomer ratios and integration values for the α-C/H correlation peaks from the major lignin interunit structures are provided on the figures.

NMR (2D ^13^C–^1^H-correlated, HSQC) spectra of cell wall material from wild-type and *pAtIRX5:AdoMetase* plants were also obtained to determine lignin composition and structure. Analysis of the aromatic region of the spectra confirmed the higher relative amount of H units in transgenics (7.3–9.7%) compared to wild type (2.7%), as well as a reduction of S units (Figure 4A). Moreover, analysis of the aliphatic region of the spectra indicated an increase of β-aryl ether (β-*O*-4) linkages and diminution of phenylcoumaran (β-5) and resinol (β-β) linkages in the lignin of transgenic plants (Figure 4B).

### Monosaccharide Composition in *pAtIRX5:AdoMetase* Lines

Monosaccharide composition was determined in mature senesced stems after sulfuric acid hydrolysis of total cell wall polysaccharides (Table 5). HPAEC-PAD and LC–MS analyses of cell wall hydrolyzates showed that biomass from *pAtIRX5:AdoMetase* lines contains less 4-*O*-MeGlcA (−72%) but more non-methylated GlcA (+ 58–60%) as well as more glucose (+ 9–13%) and mannose (+ 24–36%). Considering that GX is the main source of 4-*O*-MeGlcA and GlcA residues in cell wall biomass of mature *Arabidopsis* stems, we conclude that the *pAtIRX5:AdoMetase* lines have a lower GX methylation degree (~25–26% of total GlcA residues) compared to wild type (~75% of total GlcA residues). Moreover, higher amount of mannose along with an increase of glucose content measured in trifluoroacetic acid hydrolyzates of cell wall residues from the transgenic lines could be explained by an enrichment in hemicellulosic glucomannan (Table 5).

**Table 5:** **Chemical composition of total cell wall sugars in senesced mature dried stems from wild-type and *pAtIRX5:AdoMetase* (*AdoMetase*) lines**.

Sugar	Mean ± SE (mg g^−1^ dry weight)			
	Wild type	*AdoMetase-1*	*AdoMetase-2*	*AdoMetase-3*
Fucose	3.1 ± 0.1	3.1 ± 0.0	3.1 ± 0.0	3.1 ± 0.0
Rhamnose	7.2 ± 0.2	6.6 ± 0.1	6.5 ± 0.2	6.4 ± 0.2
Arabinose	7.8 ± 0.1	8.1 ± 0.3	7.6 ± 0.3	7.9 ± 0.2
Galactose	13.2 ± 0.4	13.5 ± 0.4	13.3 ± 0.4	13.7 ± 0.3
Mannose	22.7 ± 1.8	35.3 ± 3.2*	30.8 ± 3.2*	29.8 ± 0.9*
Galacturonic acid	61.9 ± 8.9	51.9 ± 6.9	55.6 ± 5.3	65.8 ± 1.1
Xylose	185.1 ± 12.7	220.8 ± 9.9	192.9 ± 10.0	184.2 ± 9.4
Glucose	376.4 ± 9.2	433.3 ± 13.4*	422.2 ± 15.7*	413.7 ± 13.7*
Glucose (TFA)	10.4 ± 0.3	12.9 ± 0.2*	14.3 ± 0.3*	14.2 ± 0.3*
Glucuronic acid	2.2 ± 0.1	5.2 ± 0.8*	5.2 ± 0.8*	5.6 ± 0.2*
4-*O*-Methylglucuronic acid	6.8 ± 0.8	1.9 ± 0.3*	1.9 ± 0.1*	1.9 ± 0.2*

“Glucose (TFA)” is the amount of glucose released after trifluoroacetic acid hydrolysis of cell wall residues. Values are means ± SE from three biological replicates (*n* = 3). Asterisks indicate significant differences from the wild type using the unpaired Student’s *t*-test (*P < 0.05).

### Saccharification Efficiency in *pAtIRX5:AdoMetase* Lines

**Figure 5:**
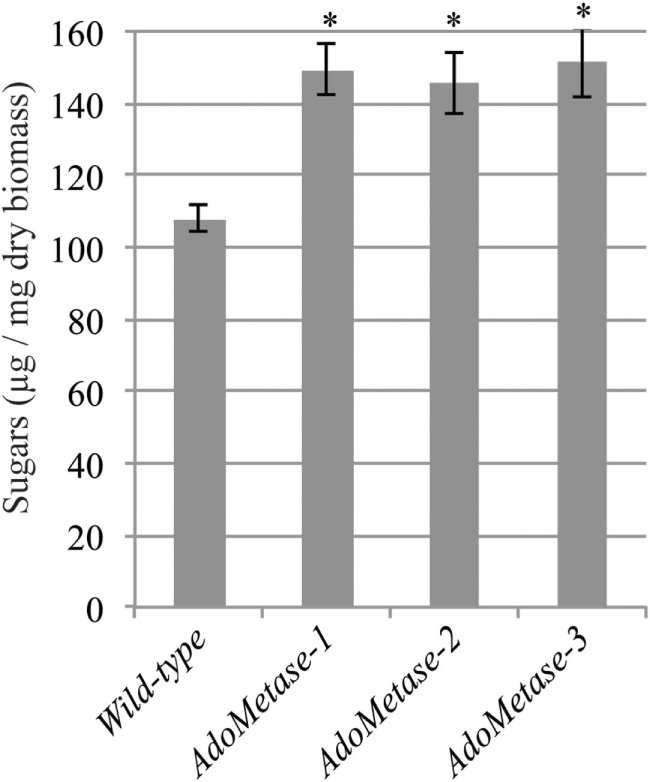
**Saccharification of biomass from mature senesced stems of wild-type and *pAtIRX5:AdoMetase* (*AdoMetase*) lines**. Values represent the amounts of sugars released from biomass after a dilute alkaline pretreatment and 48-h enzymatic digestion with cellulase cocktail (1% w/w). Values are means ± SE of six biological replicates (*n* = 6). Asterisks indicate significant differences from the wild type using the unpaired Student’s *t*-test (*P < 0.001).

Saccharification assays after dilute alkaline pretreatment of stem material were conducted to evaluate the cell wall digestibility of the *pAtIRX5:AdoMetase* lines. As shown in Figure 5, higher amount of sugars (+ 26–29%) were released from the biomass of the transgenic lines after 48 h of enzymatic hydrolysis with commercial cellulase cocktail. These data demonstrate that cell wall biomass from the *pAtIRX5:AdoMetase* lines is less recalcitrant to cellulase digestion.

## Discussion

Cellulosic biomass contains 20–30% lignin, and more than 10% of carbon stored in the lignin is derived from AdoMet. In particular, G and S lignin units, which represent more than 95% of lignin units, consist of 10 and 11 carbon skeletons, respectively, and harbor one or two methyl groups added by AdoMet-dependent methyltransferases (CCoAOMT and COMT). This makes lignin the major sink for AdoMet utilization in stem tissues developing SCWs. Here, we show that expressing AdoMetase in these tissues reduces lignin content. AdoMetase cleaves AdoMet and generates homoserine and methylthioadenosine (Figure 1B). Our metabolite analysis of plants expressing AdoMetase revealed a ~50% reduction of AdoMet pools, whereas Met content was not reduced compared to wild type (Table 2). These data suggest that AdoMet synthetase activity becomes a limiting factor and cannot compensate for AdoMetase activity to maintain AdoMet pools at the level of the wild type. Furthermore, AdoMetase generates homoserine, which can potentially be recycled into Met and threonine via *O*-phosphohomoserine (Figure 1B; Jander and Joshi, 2009). In the case of AdoMetase transgenic lines, such recycling could be occurring during Met synthesis since accumulation of homocysteine was observed, and likely takes place during the synthesis of threonine, as its content is increased threefold. For undetermined reasons, Met content was ~twofold higher in one of the AdoMetase transgenic lines, which could be due to higher AdoMetase activity, more efficient recycling of homoserine into Met, or the consequence of higher activity of one or several enzymes of the Yang cycle (① to ⑧ in Figure 1B).

In *Arabidopsis*, AdoMet content is not considered to be limiting for lignin biosynthesis (for S units in particular) and GX methylation since overexpression of ferulate 5-hydroxylase and of GX methyltransferase result in higher S-lignin units and a higher degree of GX methylation, respectively (Meyer et al., 1998; Yuan et al., 2014). Nevertheless, our data show that a ~50% reduction of AdoMet content in *Arabidopsis* stems due to AdoMetase expression leads to reductions of lignin content and degree of GX methylation. In our analysis of cell wall monosaccharides, we observed in AdoMetase transgenic lines a reduction of 4-*O*-MeGlcA which is associated with an increase of GlcA and no change in xylose content, showing that reducing the degree of GX methylation degree does not affect GX content. This has been observed previously in GX methyltransferases mutants that display various degrees of GX methylation and yet showed no differences for the content of monosaccharides (Lee et al., 2012; Urbanowicz et al., 2012; Yuan et al., 2014). These results also suggest that the increase of glucose observed in cell walls of the AdoMetase transgenic lines would be a consequence of the reduction of lignin rather than the change in GX methylation. Both CCoAOMT and COMT are predicted to be located in the cytosol for the biosynthesis of lignin (Ruelland et al., 2003; Tanz et al., 2003), whereas GX methyltransferases are localized in the Golgi apparatus for the methylation of GlcA residues (Lee et al., 2012; Urbanowicz et al., 2012), which implies that AdoMetase affects AdoMet pools in both the cytosol and the Golgi apparatus.

Lignin content is reduced by ~30% in the AdoMetase transgenic lines compared to that in wild-type plants, and the S/G ratio is decreased due to relative reduction of S units, indicating that synthesis of both G and S units is compromised. Moreover, lignin from the transgenic lines shows a relative increase of H units, which has been previously observed in lignins from plants and cell cultures affected in CCoAOMT activity (Do et al., 2007; Wagner et al., 2011). In AdoMetase transgenic plants, we also measured a lower amount of cell wall-bound ferulate, which is derived from the incorporation of feruloyl-CoA synthesized by CCoAOMT. These observations indicate that both of the methylation steps catalyzed by CCoAOMT and COMT in the lignin biosynthetic pathway are partially inhibited in transgenic lines. Nevertheless, CCoAOMT and COMT are possibly affected differently in AdoMetase-expressing lines, depending on their respective affinity for AdoMet and activity at lower intracellular AdoMet concentration. Furthermore, reduction of CCoAOMT and COMT activities has to be moderate in these lines since simultaneous disruption of both genes results in growth arrest at the seedling stage (Do et al., 2007).

We show that cell wall biomass from plants expressing AdoMetase is less recalcitrant to cellulose degradation, which probably results from changes in lignin content and structure since even a complete loss of GX methylation was shown not to affect glucose yields during saccharification (Yuan et al., 2014). Lignin is presumably less recalcitrant in the case of the AdoMetase transgenic lines because it contains higher amounts of the more labile β-*O*-4 linkages and fewer condensed C-C linkages. Moreover, an increase in the relative amount of H units typically affects the degree of polymerization of lignin and its extractability under alkaline treatment, which could promote biomass saccharification efficiency (Ziebell et al., 2010; Eudes et al., 2015).

Many metabolites other than lignin monomers require one-carbon units and AdoMet for biosynthesis, although, in these cases, the demand in SCW-producing tissues is considerably below that allocated for lignin biosynthesis (Hanson and Roje, 2001). Nevertheless, in stems of the AdoMetase transgenic lines, it would be interesting to assess the impact of AdoMetase expression on the content of metabolites derived from AdoMet such as thermospermine and ethylene, both of which have been found to be detrimental to differentiation of plant cells into vascular cells (Bollhöner et al., 2012; Takano et al., 2012). One hypothesis is to suggest that AdoMet levels are still sufficient to sustain the synthesis of these metabolites in stems of AdoMetase-expressing plants because AdoMetase is expressed under the control of the promoter of an SCW cellulose synthase gene (*IRX5*) that is active after xylem cell differentiation, when cells turn on their SCW synthesis machinery. In an observation similar to ours, mutation of FPGS1, which is preferentially expressed in vascular tissues of *Arabidopsis*, resulted in a 50% reduction of AdoMet content in stems, a 17% reduction in lignin content, and no loss of above-ground biomass (Srivastava et al., 2015).

In our view, expression of AdoMetase offers promise as a new approach for engineering bioenergy crops through modification of lignin. To optimize biomass improvement without affecting yields, such heterologous expression could be conducted with higher precision using different tissue-specific promoters that are also active at different SCW developmental stages.

## Author Contributions

AE designed and performed experiments, supervised the study, analyzed data, prepared figures, and co-wrote the manuscript; NZ, JL, and SY performed some experiments; GW performed LC/MS analysis; EB developed LC/MS methods, contributed data analysis, co-wrote the manuscript, and provided some supervision and technical support; NS performed NMR experiments, contributed data analysis, co-wrote the manuscript, and provide financial support; DL contributed data analysis, critically read the manuscript, edited the document, and provided financial support and supervision; TL, SS, JM, JK, and BS provided financial support and supervision.

## Conflict of Interest Statement

JK has financial conflicts of interest in Amyris, LS9, and Lygos. DL has financial conflicts of interest in Afingen. The remaining co-authors declare that the research was conducted in the absence of any commercial or financial relationships that could be construed as a potential conflict of interest.
