# Supplementary material for: Expression of S-adenosylmethionine Hydrolase in Tissues Synthesizing Secondary Cell Walls Alters Specific Methylated Cell Wall Fractions and Improves Biomass Digestibility
Source: Front Bioeng Biotechnol. 2016 Jul 19;4:58. doi: 10.3389/fbioe.2016.00058 (PMC4949269; doi:10.3389/fbioe.2016.00058)
Supplement: Supplementary file 1 [file table_1.docx]

| **Table S1**. Oligonucleotides used in the study. | |
| --- | --- |
| **Primer name** | **Sequence (5’-3’)** |
| Tub8-fw | GGGCTAAAGGACACTACACTG |
| Tub8-rv | CCTCCTGCACTTCCACTTCGTCTTC |
| AdoMetase-fw | CCAATCTTTGCGATGAGGTTAATATG |
| AdoMetase-rv | GTCCTGAACTTGCCACCTTCTTC |

| **Table S2.** Characteristics and relative molar abundances (%) of the compounds released after pyro-GC/MS of extractive-free senesced mature stems from wild-type (WT) and *pAtIRX5::AdoMetase* (*AdoMetase*) plants. Values in brackets are the SE from four biological replicates (*n* = 4). | | | | | | | | | |
| --- | --- | --- | --- | --- | --- | --- | --- | --- | --- |
| Compound name | Origin | Formula | Molecular mass | Main mass fragments | Elution time (min) | WT  (%) | *AdoMetase-1*  (%) | *AdoMetase-2* (%) | *AdoMetase-3* (%) |
| Phenol | H | C_6_H_6_O | 94 | 65, 66, *94* | 4.28 | 0.3 (0.1) | 0.5 (0.2) | 1.0 (0.2) | 0.6 (0.2) |
| 2-Methylphenol | H | C_7_H_8_O | 108 | 77, *107*, 108 | 4.96 | 1.0 (0.3) | 2.2 (0.6) | 2.7 (0.5) | 2.0 (0.3) |
| 3-Methylphenol | H | C_7_H_8_O | 108 | 77, *107*, 108 | 5.16 | 0.2 (0.0) | 0.5 (0.2) | 0.3 (0.1) | 0.4 (0.1) |
| 2-Methoxyphenol | G | C_7_H_8_O_2_ | 124 | 81, *109*, 124 | 5.34 | 11.1 (0.5) | 6.2 (1.1) | 8.3 (1.7) | 5.2 (1.0) |
| 2,5-Dimethylphenol | H | C_8_H_10_O | 122 | 77, 107, *122* | 5.93 | 1.2 (0.1) | 1.4 (0.3) | 2.2 (0.4) | 1.0 (0.2) |
| 4-Ethylphenol | H | C_8_H_10_O | 122 | 77, *107*, 122 | 6.15 | 0.4 (0.1) | 0.6 (0.1) | 0.8 (0.4) | 0.5 (0.1) |
| 2-Methoxy-5-methylphenol | G | C_8_H_10_O_2_ | 138 | 95, *123*, 138 | 6.45 | 12.0 (0.5) | 6.7 (1.0) | 9.7 (1.7) | 7.5 (1.4) |
| 4-Ethyl-2-methoxyphenol | G | C_9_H_12_O_2_ | 152 | 122, *137*, 152 | 7.45 | 1.1 (0.1) | 1.9 (0.6) | 1.2 (0.2) | 1.0 (0.2) |
| 4-Ethenyl-2-methoxyphenol | G | C_9_H_10_O_2_ | 150 | 107, *135*, 150 | 7.88 | 20.6 (0.5) | 15.9 (1.9) | 17.8 (2.2) | 14.8 (1.2) |
| 2,6-Dimethoxyphenol | S | C_8_H_10_O_3_ | 154 | 111, 139, *154* | 8.36 | 3.4 (0.2) | 1.5 (0.5) | 1.9 (0.8) | 1.5 (0.7) |
| 2-Methoxy-4-propenylphenol | G | C_10_H_12_O_2_ | 164 | 131, 149, *164* | 8.41 | 2.6 (0.2) | 1.6 (0.3) | 1.3 (0.2) | 1.0 (0.1) |
| 4-Hydroxy-3-methoxyphenylacetaldehyde | G | C_10_H_14_O_2_ | 166 | 122, *137*, 166 | 8.52 | 0.5 (0.1) | 0.5 (0.1) | 0.5 (0.1) | 0.3 (0.0) |
| 4-Hydroxy-3-methoxybenzaldehyde | G | C_8_H8O3 | 152 | 109, 151, *152* | 9.02 | 1.9 (0.1) | 1.2 (0.2) | 1.6 (0.4) | 1.3 (0.3) |
| 4-Methyl-2,6-dimethoxyphenol | S | C_9_H_12_O_2_ | 168 | 125, 153, *168* | 9.47 | 1.6 (0.2) | 0.6 (0.0) | 1.2 (0.4) | 0.9 (0.3) |
| 2-Methoxy-4-propenylphenol | G | C_10_H_12_O_2_ | 164 | 131, 149, *164* | 9.52 | 11.4 (0.2) | 6.3 (1.1) | 8.5 (0.9) | 5.7 (1.1) |
| 4-Hydroxy-3-methoxyacetophenone | G | C_9_H_10_O_3_ | 166 | 123, 151, 166 | 10.05 | 2.7 (0.4) | 2.0 (0.3) | 2.2 (0.7) | 1.7 (0.2) |
| 4-Ethyl-2,6-dimethoxyphenol | S | C_10_H_14_O_3_ | 182 | *167*, 182 | 10.42 | 2.5 (0.2) | 1.6 (0.3) | 1.9 (0.4) | 1.3 (0.2) |
| 4-Hydroxy-3-methoxyphenyl acetone | G | C_10_H_12_O_3_ | 180 | 122, *137*, 180 | 10.56 | 3.1 (0.5) | 2.0 (0.5) | 2.2 (0.3) | 1.9 (0.4) |
| 4-Hydroxy-3,5-dimethoxystyrene | S | C_10_H_12_O_3_ | 180 | 137, 165, *180* | 10.88 | 16.7 (0.3) | 6.4 (1.4) | 7.6 (1.0) | 5.3 (1.1) |
| 4-Allyl-2,6-dimetoxyphenol | S | C_11_H_14_O_3_ | 194 | 167, 179, *194* | 11.32 | 0.0 (0.0) | 0.0 (0.0) | 0.1 (0.1) | 0.1 (0.1) |
| 4-Hydroxy-3,5-dimethoxybenzaldehyde | S | C_9_H_10_O_4_ | 182 | 167, 181, *182* | 12.07 | 0.0 (0.0) | 0.0 (0.0) | 0.0 (0.0) | 0.0 (0.0) |
| 4-Propinyl-2,6-dimethoxyphenol | S | C_11_H_12_O_3_ | 192 | 106, 131, 177, *192* | 12.23 | 0.0 (0.0) | 0.0 (0.0) | 0.0 (0.0) | 0.0 (0.0) |
| 4-Propenyl-2,6-dimethoxyphenol | S | C_11_H_14_O_3_ | 194 | 167, 179, *194* | 12.43 | 3.0 (0.5) | 2.6 (0.9) | 0.4 (0.4) | 1.5 (0.5) |
| 4-Hydroxy-3,5-dimethoxyacetophenone | S | C_10_H_12_O_4_ | 196 | 153, *181*, 196 | 12.88 | 0.9 (0.3) | 0.4 (0.0) | 0.8 (0.2) | 0.8 (0.4) |
| 4-Hydroxy-3-methoxycinnamaldehyde | G | C_10_H_10_O_3_ | 178 | 107, 135, 147, *178* | 13.00 | 0.0 (0.0) | 0.0 (0.0) | 0.0 (0.0) | 0.0 (0.0) |
| 4-Hydroxy-3,5-dimethoxyphenylacetone | S | C_11_H_14_O_4_ | 210 | 123, *167*, 210 | 13.26 | 1.6 (0.4) | 0.5 (0.0) | 0.8 (0.3) | 0.4 (0.1) |
| 4-Hydroxy-3,5-dimethoxyphenylethanone | S | C_10_H_12_O_4_ | 196 | 153, *181*, 196 | 13.83 | 0.0 (0.0) | 0.0 (0.0) | 0.0 (0.0) | 0.0 (0.0) |
| % H-units |  |  |  |  |  | 3.2 (0.4) | 8.2 (0.3) | 9.1 (1.1) | 8.1 (0.8) |
| % G-units |  |  |  |  |  | 67.1 (1.2) | 70.7 (1.2) | 70.9 (1.1) | 71.1 (1.3) |
| % S-units |  |  |  |  |  | 29.7 (1.1) | 21.1 (1.1) | 20.0 (1.4) | 20.7 (1.5) |
