# Supplementary material for: Expression of S-adenosylmethionine Hydrolase in Tissues Synthesizing Secondary Cell Walls Alters Specific Methylated Cell Wall Fractions and Improves Biomass Digestibility
Source: Front Bioeng Biotechnol. 2016 Jul 19;4:58. doi: 10.3389/fbioe.2016.00058 (PMC4949269; doi:10.3389/fbioe.2016.00058)
Supplement: Supplementary file 2 [file data_sheet_1.docx]

Codon-optimized nucleotide sequence encoding AdoMetase from enterobacteria phage T3 (UniProtKB/Swiss-Prot accession number P07693.1) flanked with Gateway *att*B1 (5’-end) and *att*B2 (3’-end) recombination sites (in blue):

GACAAGTTTGTACAAAAAAGCAGGCTTCATGATCTTCACCAAGGAGCCAGCACACGTTTTCTATGTTTTAGTTTCAGCATTCAGGTCCAATCTTTGCGATGAGGTTAATATGTCCAGACATAGGCACATGGTTTCTACTCTTAGAGCTGCACCAGGTTTGTATGGAAGTGTGGAATCCACCGATTTGACTGGTTGTTACAGGGAGGCTATTTCTTCAGCACCTACAGAAGAGAAAACCGTTAGAGTGAGGTGCAAAGATAAGGCTCAAGCATTGAATGTTGCTAGATTAGCATGTAACGAATGGGAGCAAGACTGCGTTTTAGTGTATAAGTCTCAGACTCATACAGCTGGTCTTGTTTATGCAAAAGGTATTGATGGATACAAGGCTGAAAGATTGCCAGGATCATTTCAAGAGGTGCCAAAGGGTGCTCCTTTACAGGGATGTTTTACAATCGACGAATTCGGAAGAAGGTGGCAAGTTCAGGACCCAGCTTTCTTGTACAAAGTGGTCTGAGACCCAGCTTTCTTGTACAAAGTGGTC
